# Protected areas and poverty

**DOI:** 10.1098/rstb.2014.0271

**Published:** 2015-11-05

**Authors:** Daniel Brockington, David Wilkie

**Affiliations:** 1 Conservation and Development, IDPM, University of Manchester, Manchester, M13 9PL, UK; 2 Conservation Measures, Wildlife Conservation Society, 2300 Southern Boulevard, Bronx, NY 10460, USA

**Keywords:** conservation, parks, well-being, livelihoods, displacement, environmental regulation

## Abstract

Protected areas are controversial because they are so important for conservation and because they distribute fortune and misfortune unevenly. The nature of that distribution, as well as the terrain of protected areas themselves, have been vigorously contested. In particular, the relationship between protected areas and poverty is a long-running debate in academic and policy circles. We review the origins of this debate and chart its key moments. We then outline the continuing flashpoints and ways in which further evaluation studies could improve the evidence base for policy-making and conservation practice.

## 1. Introduction

Protected areas are controversial. To many they are essential because their restrictions on natural resource use conserve biological systems that will otherwise be depleted, degraded or destroyed. To critics protected areas threaten peoples' rights and livelihoods, allowing access for some people but excluding others. Protected areas' distribution of fortune and misfortune lies at the heart of their controversies.

The controversies are complicated in two ways. First, protected areas are written into the founding stories that nations tell about themselves [1]. They are attributes of modernity, progress and development; they matter to governments. At a more personal scale, the beauty of protected areas is written into many of our memories and aspirations for a good life. These connections to our national and self-identities mean that the very idea that parks could be controversial is itself controversial.

Second, the local consequences of protected areas can be highly contrary. There is evidence that people have been displaced or denied access to resources by the establishment of parks and reserves [2,3]. Conservation has caused poverty because of the restrictions of protected areas [4]. Yet protected areas have also benefitted peoples' livelihoods [5], and secured the rights of people to land and valuable natural resources that they risked losing to more powerful groups, firms or the state [6–9]. In one case, the removal of people resulted in *greater* levels of equality and well-being [10]. The diversity of cases is captured in a growing literature, with conceptual analyses of the relationships between poverty and conservation [11,12]. Reviews of this literature are available in conservation and anthropology journals [13,14], including two systematic reviews [15,16]. There are also edited collections on the topic [17,18].

This essay builds on two premises: that protected areas are requisite for effective conservation, and that their existence and creation will distribute misfortune and fortune. From these starting points, we map the ongoing tensions that characterize debates about protected areas. We highlight issues that will likely emerge in the future and examine how high-quality evaluations can contribute to addressing them. We first provide a short history of the sometimes acrimonious debate between advocates and critics of protected areas. We then briefly review the main issues that make this debate contentious. Lastly, we offer ways forward in the hope that we can help depolarize the debate, and find common ground on the roles and values of protected areas that take seriously the interests of rights holders.

## 2. A brief outline of the parks and people debate

There are three components to contests about parks and people. One element is historical. It follows a re-awakening of interest in the possibility that parks can cause their neighbours difficulties. Historians have emerged from the archives of national parks services and conservation organizations with alarming stories of violence, eviction and dispossession [19–22]. Such stories can be found in the very places where John Muir urged his compatriots to let ‘nature's peace … flow into you as sunshine flows into trees … while cares will drop off like autumn leaves [23, p. 56]’. Parks, popular exemplars of tranquillity, seem also to be a source of injustice, and the instrument behind the alienation and exclusion of diverse indigenous people and the rural poor. A sub-genre of history, geography and anthropology now explores the different ways protected areas are built upon histories of exclusion.

A second element is conceptual. It questions what sorts of nature parks conserve. Some protected areas are envisaged to protect untrammelled nature from human interference—yet that very nature is itself the product of particular types of interaction with people [24–26]. Wilderness areas, places without people, have been created despite the role of people in their history and ecology. To make matters worse, the new managers of these wildernesses then set about doing the most unnatural things (suppressing fire, removing predators) and replacing one anthropogenic landscape with another, all in the name of wilderness preservation [27]. This history does not mean that wild places do not exist, that they are not threatened and that their persistence is not important [28–30]. But it does question some of the ideology and motivations underlying the call for creating and managing protected areas.

The third element is the broader politics surrounding conservation, land use and livelihoods, of which contests over protected areas are part. These disputes include contests over traditional territorial claims, land-grabbing by the landless or industrial-scale commodity producers, as well as evictions and exclusion from protected areas. They tend to be bound up in bigger national debates over citizens' individual and collective rights. For example, tensions between highlanders and lowlanders in Thailand have a long history. Lowlanders have tried to keep highlanders out of the forests for fear that forest use will disrupt water supplies needed in lowland rice cultivation [31]. In Tanzania, tensions between pastoralists and conservationists are part of an older conflict with the state about how to control and develop unruly pastoral populations [32]. More recent manifestations of these conflicts concern the alliances of corporate capital and conservation and the subsequent possibilities for ‘green-grabbing’, which add to the existing problems of land-grabbing [33].

These three elements have combined in a slowly evolving debate that involves both collaborations and confrontations between conservationists and human interests. The collaborations date from at least the 1980s in initiatives to make conservation more profitable, or at least not costly, for local people. They included attempts to allow local people to control natural resources directly and benefit from revenues generated from sustainable uses such as wildlife viewing or hunting. These attempts tried to produce conservation with ‘a human face’ [34]. In the 1990s, the interests of indigenous groups and conservationists converged as both realized that each could help the other exclude unwelcome development of wilder landscapes [4,35].

But there are also tensions in these alliances. Some conservationists felt, and still feel, that attempts to engage in development activities are a difficult and an unwelcome distraction [36–39]. Their core business is conservation, not poverty reduction [40,41]. Others argued they were not primarily interested in conserving the hunted landscapes that indigenous groups wanted to protect [42,43]. Nonetheless, a small but growing number of conservation organizations argue that securing local livelihoods is essential to the long-term success of protected areas. They see enhancing livelihoods as a purposeful strategy to achieve conservation goals by providing tangible incentives. They also view it as an ethical obligation to ensure that poor local families are not shouldering the cost of conserving a global public good [44]. At the same time, human rights and indigenous peoples activists, as well as social scientists, were uncovering cases of disadvantage and impoverishment resulting from conservation measures [18,45].

Many of the tensions that had been simmering within the conservation movement, and between it and its critics [46], were loudly and publically brought to a head at the World Parks Congress of 2003 in Durban. At this meeting, the International Union for Conservation of Nature (IUCN) announced that 10% of the land surface of the planet had been conserved in protected areas. The announcement was mistaken—that target had been achieved shortly before it was set, back in 1992, but the improved World Database of Protected Areas now made it possible to realize how much land was legally designed as protected [14]. This milestone divided participants at the conference. Most were delighted, but remained concerned that so much threatened land and so many types of ecosystem (particularly marine) remained unprotected. But a number feared that expansion of protected areas would simply exacerbate the ills to local livelihoods known to be, at times, a consequence of conservation.

The tone of debates was a surprise to many conservationists [47]. It was strange that participants at a World Parks Congress should not want to celebrate the advance of their cause. It was stranger still that there was protest against conservation at one of the global movement's show piece events. But things were about to get even more fiery. In 2004, Mac Chapin published a polemical paper that alleged that three major conservation organizations were intimately involved in processes that disadvantaged local groups and were allying with large corporate interests to do so [48].

Since these rancorous public disagreements, the debate has become generally less heated. A number of conservation organizations have taken the charges seriously, resulting in thoughtful reviews of the problem [49]. Others are hiring more social scientists to help avoid or mitigate potentially adverse human livelihood impacts of conservation. For their part, critics of conservation are setting conservation conflict in broader settings, as part of the machinations of broader political economic forces and the workings of capitalism itself [4,50,51].

## 3. Flashpoints in the protected area debates

Though the character of the debate has changed, there remain four persistent flashpoints that continue to cause friction. Two of these flashpoints concern the precise nature of the problems protected areas are thought to cause. First, there is the question of what form of displacement matters, physical or economic. Physical displacement, or eviction, entails the involuntary removal of people from their homes and homelands. Economic displacement refers to restrictions that make it hard to pursue a livelihood [52,53]. One might be allowed to live in a national park, but not allowed to cut thatching grass or firewood, or plant crops and graze livestock. This is a flashpoint because attention is often focused on eviction. Physical displacement, after all, is the most painful thing a state (with the right of eminent domain) can do to its law-abiding citizens. Economic displacement typically inflicts a lower cost, and thus is less visible and shocking. However, Brockington and Igoe's review of eviction for conservation suggested that economic displacement is actually more prevalent than eviction and is also less likely to result in appropriate compensation. They concluded that attention given to rare cases of physical displacement was distracting from the more common problem of economic displacement [54].

Second, there is the quality of data employed by both sides of the debate. Indeed we, the two authors, have disagreed over the evidence with respect to eviction. Wilkie and colleagues have argued that ‘to date little empirical evidence exists to substantiate the contention that parks are bad for local people’ ([55], p. 247) and Brockington (with Igoe) has argued that there was evidence of eviction from over 180 protected areas (while noting that the quality of evidence about the consequences of those evictions was often poor) [54]. Similarly, there have been disputes over the scale of impacts. Some authors have claimed that conservation correlates directly with national-level poverty and that protecting more land makes people poorer [56–58]. Other authors dismiss the claim, asserting that the local impacts of protected areas are just that: local [59]. The opposite claim, that living next to a protected area is so beneficial that one can detect migration to their boundaries, has been dismissed as deriving from inappropriate use of population databases [60–62]. Fierce disputes in the literature also contest the quality of particular individual and regional case studies [63,64].

The third flashpoint concerns who is entitled to consideration and compensation related to eviction or displacement. Many of the most vocal and vituperous disputes about evictions and displacement have focused on indigenous people, who are generally viewed as deserving consideration and compensation. By contrast, being classified as ‘not indigenous’ places even legitimate rights holders and important stakeholders at a disadvantage [65]. Goldman recounts the efforts the World Bank made to avoid relocating indigenous people, by having them reclassified as not indigenous [66]. In classic cases such as Mkomazi and the Ngorongoro Crater Area (both in Tanzania), there were vigorous disputes as to who could be called the rightful residents of the area, and should be allowed to remain, despite the presence of conservation restrictions [32,67]. Similarly people who are not indigenous make vigorous and often justifiable claims to resource and property rights. The most well-publicized example of this is the example of the rubber tappers of the Brazilian Amazon—people of mixed heritage who relocated to their current lands only in the past century [68]. Likewise, on Sibuyan Island, Philippines, WWF worked with the indigenous Sibuyan Mangyan Tagabukid to gain title to lands that overlap with Mount Guiting-Guiting Natural Park. This resulted in the alienation of the rest of the island's inhabitants, who felt they had been cheated out of access to land and livelihood opportunities [69].

A final flashpoint concerns the governance of protected areas, and whether local or state authority is more effective for generating conservation outcomes and local prosperity. Devolution of management authority has been portrayed as a form of community conservation that could replace traditional protected areas that are established and managed by the state [70–72]. Proponents of devolution and the principle of subsidiarity point out that private land owners and communities with traditional claims over territory have long-established *de facto* protected areas. Moreover, capacity at the national level to enforce protected area regulations is often weak, as is state willingness or ability to compensate adequately people subject to physical or economic displacement. However powerful devolution may be in theory, it has proved difficult in practice [73]. Communities are riven with tensions and divisions, with incompetent or corrupt leadership [74]. Sometimes devolution is proscribed, or authority delegated to individuals or groups that are not downwardly accountable, transparent in their decision-making, or even competent to manage resources well. Sometimes authority is devolved to groups who may wish to liquidate the natural resources they live with, to pursue more modern lifestyles [75].

## 4. Contributions of future studies

Existing explorations of protected areas impacts have tended to use two sorts of methodologies. Early work involved studies of individual protected areas and entailed surveys of affected human populations combining quantitative methods with qualitative work (in-depth interviews and oral histories) [10,32]. These provide some idea as to how fortune and misfortune have been locally distributed by conservation policies, but little idea as to how generalizable these findings are to other protected areas, where different livelihoods and local politics pertain. This makes it harder to understand *how* different forms of conservation protection more generally affect people.

More recently, spatially aggregated data have been used to explore the relationship between distributions of protected areas and distributions of poverty [5,76–78]. These have the advantage of being able to control for the effects of isolation and lack of infrastructure that can cause poverty, but are not necessarily a consequence of conservation policy, as well as capturing the local multiplying economic consequences that forces like tourism can produce. However, because they use spatially aggregated data, they are weaker at portraying the smaller scale distribution of fortune and misfortune around protected areas. This weakness, combined with the fact that we cannot infer cause from spatial correlation without very strong, untestable assumptions, makes understanding causal relationships hard.

Reviews of this work do not necessarily solve the problems of understanding causes because they have to take a scattergun approach, using studies from all over the world that fulfil their quality criteria [15]. This can entail comparing protected areas in Norway, Sweden, Mexico and Thailand (for example), which exist in completely different political, economic and socio-historical contexts. It is difficult to make such comparisons robustly.

Clearly, one of the priorities for further evaluations is ways of better understanding the causal mechanisms behind the impacts of protected areas. There are some useful interventions. Miteva *et al*. provide a roadmap of the ways in which large-scale quantitative comparisons could begin to identify causal mechanisms of different forms of biodiversity policy, including protected areas [79]. Ferraro & Hanauer [80] have applied some of these techniques to Costa Rica, observing that tourism appears to explain most of the observed poverty reduction. This work (which used spatially aggregated data) still leaves open the question of how such benefits are distributed within affected groups. However, other techniques and studies that explore distributional aspects on large scales can be used to elucidate this aspect [81–83].

One important issue, which is not well considered, is how different forms of protection produce different outcomes for people. Further evaluation studies could explore the link between governance regime (state, co-management, community, etc.), protected area category, protection practices and the negative or the positive impacts of protected areas on human well-being. This type of enquiry is being conducted with respect to the outcomes of different sorts of governance for conservation effectiveness, with a common finding being that greater protection (on paper) is not necessarily the cause of better conservation outcomes [76,84]. Oldekop *et al*.'s research, based on published reviews of 160 protected areas, suggests that protected areas that enhance human well-being (by permitting sustainable use) also tend to be correlated with better conservation outcomes [16]. This was a global review, and clearly the next step would be to explore how these findings vary in different parts of the world, according to the form of sustainable use allowed and the manner of its governance.

Another area that could be usefully evaluated would be the form of compensation that works in different contexts. If compensation for loss of property or use rights is deemed appropriate, how should the eligible party be compensated and what conditions, if any, should be linked to such compensation? In cases of lost property, such as when a lion takes a rancher's cow, compensation would most likely be a one-off payment. However, if compensation were due because the eligible party was involuntarily displaced from their home to a new location, should that compensation be provided as a one-off payment or as an annuity paid annually on condition that the eligible party does not move back from whence they were evicted? In cases where a taking of rights requires a change in an individual's behaviour (e.g. when a hunter is no longer legally allowed to harvest wildlife within what was his hunting domain but is now a protected area), should compensation be made in installments, conditioned on compliance with the rights restrictions? Unless such a conditional payment system is in place, individuals may simply ignore the rights restrictions and continue using the resources as before. When compensation payments are made both to cover the costs of lost rights, and to ensure compliance with these rights restrictions, they are ostensibly direct payments for conservation. Though direct payments have been advocated as the most targeted way to effect conservation [85–87], others have labelled them as unethically coercive (i.e. forcing people to conserve) or fiscally unsustainable. Yet, in the developed world, direct payments are becoming commonplace, are lauded for their effectiveness and are not typically branded as being coercive or unsustainable.

A variety of methods will be necessary to undertake these evaluations. Pullin *et al.* [15], after their systematic review, were keen to advocate matched-control quasi-experiments and replication properly to understand the impacts of protected areas. But not all the consequences of protected areas can be captured and measured through the matched controls and replication that the authors of that paper advocate. The experience of eviction and exclusion is not well captured by such positivist frameworks. If we are to understand the issues of belonging, history, identity and security that are all integral to well-being, these will require more qualitative methods. Both Baylis *et al.* [88] and Woodhouse *et al.* [89] argue strongly for future impact evaluations to be based on clear, multidisciplinary theories-of-change that use both qualitative and quantitative approaches.

Finally, it is also interesting to consider why there are still too few systematic, timely and publicly available evaluations of conservation policies such as protected areas. Wilkie & Ginsberg [90] posited that the present model (evaluation by the conservation practitioners themselves) is flawed because, unlike public health and education academics, the incentive structure for conservation academics to participate in these evaluations is not in place. Academics have few incentives to participate because prestige journals rarely publish evaluations of conservation project effectiveness, and donors seldom fund conservation academics to conduct systematic evaluations of conservation project effectiveness. If top-tier conservation journals were to dedicate space to such evaluations, then this would enable conservation academics to benefit professionally from conducting conservation evaluations. This would match the current push in UK academia for more impact-orientated research—i.e. work that can show it is making a difference.

## 5. Conclusion

Most people would argue that protected areas are essential, and that without political support at local, national and international levels their existence is in jeopardy. Concerns about the legitimacy and desirability of protected areas come from both within and outside the conservation community. They are fuelled by conflicting expectations of what parks and reserves can and cannot, or should and should not, do.

We believe strongly that building and maintaining a robust multi-level constituency for protected areas requires honesty when characterizing their benefits and costs, and a readiness by those who reap the benefits to compensate those who incur the costs. We have outlined a number of areas where further systematic evaluation, review and research could shed light on an important debate. This is not a theoretical discussion, for in many parts of the world there are constituencies agitating for the dismantling of protected areas in the name of local people and poverty alleviation, and others where conservation restrictions are sought that can only be harmful to those people (and of questionable conservation benefit). We hope that by provoking discussion of these issues early in the debate we can avoid some of the rhetorical entrenchment that has unnecessarily polarized attitudes towards protected areas.
